# Chip-Scale Aptamer Sandwich Assay Using Optical Waveguide-Assisted Surface-Enhanced Raman Spectroscopy

**DOI:** 10.3390/nano14231927

**Published:** 2024-11-29

**Authors:** Megan Makela, Dandan Tu, Zhihai Lin, Gerard Coté, Pao Tai Lin

**Affiliations:** 1 Department of Materials Science and Engineering, Texas A&M University, College Station, TX 77843, USA; 2 Center for Remote Health Systems and Technologies, Texas A&M University, College Station, TX 77843, USA; 3 Department of Biomedical Engineering, Texas A&M University, College Station, TX 77843, USA; 4 Department of Electrical and Computer Engineering, Texas A&M University, College Station, TX 77843, USA

**Keywords:** surface-enhanced Raman spectroscopy (SERS), optical waveguides, aptamer, nanoparticles (NPs), biosensors

## Abstract

Chip-scale optical waveguide-assisted surface-enhanced Raman spectroscopy (SERS) that used nanoparticles (NPs) was demonstrated. The Raman signals from Raman reporter (RR) molecules on NPs can be efficiently excited by the waveguide evanescent field when the molecules are in proximity to the waveguide surface. The Raman signal was enhanced by plasmon resonance due to the NPs close to the waveguide surface. The optical waveguide mode and the NP-induced field enhancement were calculated using a finite difference method (FDM). The sensing performance of the waveguide-assisted SERS device was experimentally characterized by measuring the Raman scattering from various RRs, including 4-mercaptobenzoic acid (4-MBA), 5,5′-dithio-bis-(2-nitrobenzoic acid) (DTNB), and malachite green isothiocyanate (MGITC). The observed Raman spectral features were identified and assigned to the complex vibrational modes associated with different reporters. A low detection limit of 1 nM was achieved. In addition, the device sensing method was applied to the detection of the biomarker cardiac troponin I (cTnI) using an aptamer sandwich assay immobilized on the device surface. Overall, the optical waveguides integrated with SERS show a miniaturized sensing platform for the detection of small molecules and large proteins, potentially enabling multiplexed detection for clinically relevant applications.

## 1. Introduction

Raman spectroscopy is a precise analytical method that excites the vibrational modes of molecules when they interact with an excitation laser and create inelastic scattering. Raman techniques are non-destructive and generate a characteristic vibrational spectrum, thus providing insight into and information about molecular structures, chemical bonding, and environmental conditions [1]. However, the technique is limited by the weak spontaneous Raman scattering signals, thus lacking capability in low-concentration molecule detection, often requiring bulky and expensive optical components, and is thus a challenge for miniaturization from a bench-top system to a wafer-scale device [1,2].

Surface-enhanced Raman spectroscopy (SERS) provides a highly enhanced signal (10^4^ to 10^8^) compared to spontaneous Raman spectroscopy, where in SERS the target molecule is on or near a metal nanoparticle surface [3]. Because of the significant Raman enhancement, SERS provides high sensitivity suitable for tracing low concentration molecules [4]. In addition, the vibrational modes displayed in SERS spectra are fundamentally sharp and yield narrow spectral features. These characteristics allow SERS to be a platform for multi-analyte detection [5,6,7]. Because of its higher sensitivity, SERS has been researched for a wide range of applications, including healthcare [8,9,10], food safety [11,12,13,14], environmental monitoring [15,16], and hazardous material detection [17,18]. Compared to other SERS platforms, the introduction of on-chip optical waveguides for SERS detection has the potential to further improve sensitivity and reproducibility while reducing the size of the device. The dominant mechanism contributing to SERS is the increase in electromagnetic (EM) field near the plasmonic structures by excitation of the localized surface plasmon resonances, and an on-chip optical waveguide can create an evanescent field propagating along the waveguide surface [19]. By placing plasmonic NPs within the waveguide evanescent field, significant field enhancement can be obtained. Simulations by Gu et al. suggested an improvement of over 1000 times [20]. Moreover, on-chip optical waveguides have the potential to provide additional improvement in the collection and guiding of Raman scattered light [21,22]. On-chip optical waveguides use robust, low-loss materials and reliable lithographic fabrication in combination with selective wet chemical or dry plasma etching techniques to provide the opportunity for low-cost, mass-producible, microscope-less portable lab-on-chip sensing [23]. Developing a waveguide-assisted SERS (WERS) platform contributes toward fully integrated on-chip sensors with compact instrumentation, which is important to fill the gap for point-of-use applications, especially for low-concentration molecule detection requiring high sensitivity. Experiments thus far have demonstrated the concept of WERS for the detection of small molecules, such as monolayer reporter 4-nitrothiophenol (4-NTP) [21,22,24] and nanoparticle-adsorbed 4-MBA dye [20]. Limited studies used the concept for detecting large molecules like proteins; one study used a dye-labeled antibody for the detection of IgG [25]. The development of WERS for more clinically relevant large molecules is essential but is currently under-investigated in the field.

The protein cardiac troponin I (cTnI) has been well established as a blood cardiac biomarker, indicated by the American Heart Association and American College of Cardiology guidelines as the preferred marker for diagnosis of myocardial infarction (MI) [26,27]. The clinical cut-off concentration of cTnI for MI diagnosis ranges from 0.1 to 1.5 ng/mL [28,29]. Optical biosensors utilizing microfluidic chips and paper-based assays have been demonstrated for the detection of cTnI for point-of-care (PoC) applications [30,31,32,33]. Though paper-based systems offer the advantages of low cost, abundant materials, and simple disposal, they are limited in terms of long-term stability [34,35]. In addition, the use of paper-based assays is less integrated than an on-chip waveguide-assisted platform, which has the potential to incorporate both protein recognition and optical signal transduction. Notably, the recognition elements in most of these biosensors are antibodies specific to cTnI. Instead of using an antibody as the recognition element, the use of aptamer-based recognition elements improves the stability compared to antibody capture and detection reagents [36]. Nucleic acid aptamers have emerged as a promising alternative, providing similar selectivity and binding affinity [37,38] with the additional benefits of simpler synthesis, longer shelf life, lower cost, and tolerance to a wider pH and temperature range [39,40].

**Figure 1:**
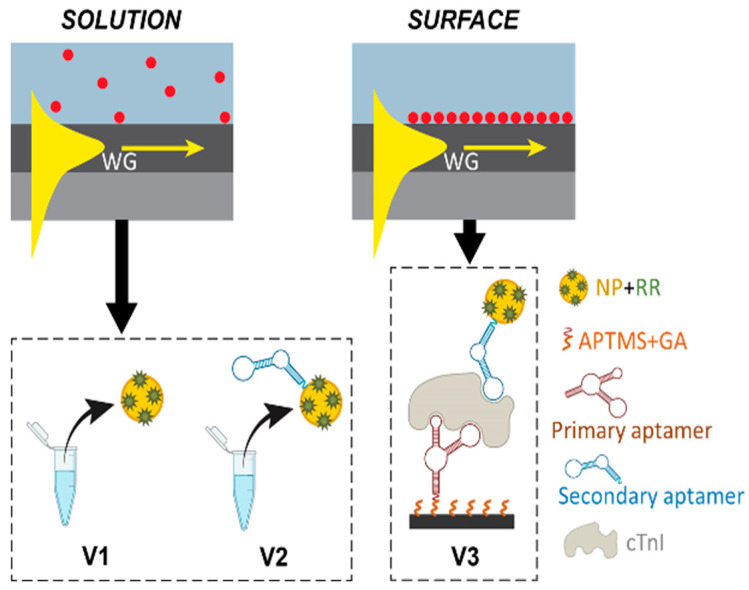
Summary of the validation steps performed using waveguide-assisted SERS detection. **V1** used the waveguide device and the colloidal NPs to detect the SERS signal from the Raman reporters in solution. **V2** used the waveguide device to detect the signal from the free-floating aptamer-conjugated RR-NP in solution. **V3** utilized the immobilized primary aptamer to trap the aptamer-conjugated RR-NP on the waveguide surface for cTnI detection. NP: nanoparticle. RR: Raman reporter.

In this work, we present the development of a SERS platform that integrated a low-refractive-index on-chip optical waveguide with colloidal NPs and demonstrated an aptamer sandwich assay for the detection of cTnI. To the best of our knowledge, this is the first research that integrates a waveguide-assisted on-chip SERS system utilizing aptamer sandwich binding for large molecule detection. Figure 1 illustrates the three experimental verification phases, **V1**, **V2**, and **V3**. In phase **V1**, the NP solutions with various small Raman reporter molecules were applied onto the surface of the waveguide device. The waveguide’s evanescent field excited the plasmon resonance of the metallic NPs that came near the waveguide surface, thus resulting in an enhanced localized electric field and creating strong Raman scattering. Here, the noble metallic NPs were prepared and mixed with a variety of Raman reporter (RR) molecules—including 4-mercaptobenzoic acid (4-MBA), 5,5′-dithiobis(2-nitrobenzoic acid) (DTNB), and malachite green isothiocyanate (MGITC). The capability of the waveguide-assisted SERS system to resolve peaks in the spectra for each of the reporter molecules was characterized. A concentration curve for MGITC in the NPs was established by correlating the waveguide-measured SERS intensity with the characteristic peaks of the assigned vibrational modes. A quantitative evaluation of the detection limit of the reporter molecules on the waveguide-assisted SERS system was carried out. Subsequently, in **V2 and V3**, aptamers were introduced to recognize and bind to the target protein cTnI. In **V2**, the RR-labeled NPs conjugated with the secondary aptamer were first tested to assess the generated SERS signal. In **V3**, aptamer sandwich binding was tested on the waveguide. The primary aptamer was immobilized on the waveguide surface using 3-aminopropyltrimethoxysilane (APTMS) and glutaraldehyde (GA). The sandwich binding between the aptamer-conjugated RR-NP, the target cTnI protein, and the primary aptamer immobilized on the waveguide enabled the signal change according to the protein. The result illustrated the potential for a miniaturized sensing device to detect clinically relevant biomolecules.

## 2. Materials and Methods

### 2.1. Materials and Reagents

Hydroxylamine hydrochloride, sodium hydroxide, silver nitrate, DTNB, and 4-MBA were purchased from Sigma Aldrich (MO, USA). MGITC was purchased from Thermo Fisher Scientific (Waltham, MA, USA). Milli-Q ultrapure water (18.2 MΩ cm^−1^) was used in all the procedures. The aptamers were synthesized by Integrated DNA Technologies (Coralville, IA, USA). cTnI was purchased from GenScript (Piscataway, NJ, USA). APTMS and GA (50 wt% solution in water) were purchased from Sigma Aldrich (St. Louis, MO, USA). All other reagents and solvents were laboratory grade or better and used as received unless otherwise stated.

### 2.2. Waveguide Sensing Device Fabrication

Optical waveguides were fabricated using a lift-off process with lithography and deposition steps. Negative tone photoresist NR9-600PY was patterned onto a 3 μm thermal silicon dioxide (SiO_2_) substrate to define the structure of the waveguides. Next, direct current (DC) reactive sputtering (PVD 75, Kurt J. Lesker) was used to deposit an aluminum nitride (AlN) thin film. The target used for sputtering material was a 4-inch diameter Al (99.999%) from Kurt J. Lesker. In a pre-sputtering process, pure argon was introduced into the chamber for 15 min to remove oxidation and clean the target surface. For the physical deposition process, argon and nitrogen were injected into the chamber in a ratio of 78% Ar and 22% N_2_, maintaining a working pressure of 3 mTorr. A deposition rate of 0.6 nm/min was achieved using 500 W of applied power. Deposition of the AlN thin film was followed by the lift-off process, leaving 600 nm tall strip waveguides with smooth surfaces and edges on the SiO_2_ undercladding. A comparable resin material was used with a rapid fabrication process to create additional optical waveguides for verification. With similar refractive indices of approximately **n_WG_** ≈ 2.0, both the dielectric and polymer waveguide material can efficiently guide the excitation light and create strong interaction with metallic NPs present in the waveguide’s upper-cladding region.

### 2.3. Nanoparticle Synthesis and NP-on-Waveguide Characterization

In step V1, the waveguide device and the colloidal NPs were used to detect the SERS signal from the Raman reporters in solution. Spherical silver nanoparticles (AgNPs) were used in V1 as the plasmonic material because of their ease of fabrication, as well as their strong interaction with light that created a large scattering cross-section [41,42]. Raman active compounds 4-MBA and DTNB are highly polarizable molecules that yield distinct and easily identifiable Raman spectra and thus are commonly used as Raman reporter molecules. A synthetic non-fluorescent dye compound, MGITC, provides absorption in the visible range used for Raman spectroscopy while avoiding the overwhelming fluorescence found with other conventional dye molecules. The absorption maximums of 4-MBA, DTNB, and MGITC were found at λ = 271 nm, 402 nm, and 620 nm [43], respectively, which rank from farthest to closest to the 635 nm Raman excitation laser line.

The AgNPs were synthesized using a “cold” method. Initially, 1 mL of 150 mM hydroxylamine hydrochloride (150 mM) was mixed with 89 mL of 3 mM sodium hydroxide solution. Secondly, 10 mL of 10 mM silver nitrate solution was added to the mixture under vigorous stirring. Finally, the mixture was left stirring for 15 min at room temperature to finish the synthesis of the AgNPs.

Following synthesis, the colloidal AgNPs were used on the waveguide device to detect the signal from the Raman reporters in solution. The following mixtures were prepared for this test. The AgNPs were mixed with one of three Raman reporter molecules: DTNB, 4-MBA, or MGITC. To create a final concentration of 0.5 μM MGITC, 100 μL of 5 μM MGITC solution was added to 1 mL AgNPs. A volume of 100 μL of 10 mM DTNB stock solution was added to 1 mL AgNPs to yield a final concentration of 1 mM DTNB. A solution of 1 mM 4-MBA in 1 mL AgNPs was prepared in a similar manner. Additionally, varying concentrations of MGITC in AgNPs (1 nM, 2 nM, 2.5 nM, 5 nM, 10 nM, 25 nM, 50 nM) were made by adding different volumes of 10 μM MGITC (0.1 μL, 0.2 μL, 0.25 μL, 0.5 μL, 1 μL, 2.5 μL, 5 μL) to 1 mL AgNPs, respectively.

### 2.4. Waveguide-Assisted cTnI Assay

**Figure 2:**
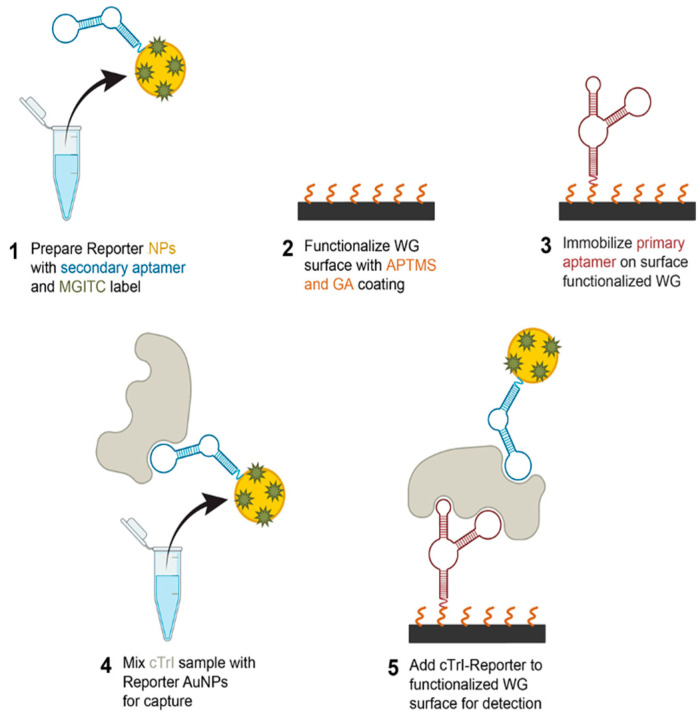
Illustration showing the preparation steps and the components of the aptamer sandwich assay that detected the biomarker cTnI using a waveguide (WG)-assisted SERS device.

A waveguide-assisted SERS assay was developed for the detection of cTnI, as illustrated in **V3**. The design of the assay was an aptamer-based sandwich assay, previously demonstrated on a paper-based sensing system [44,45]. These aptamer sequences have shown sensitive and selective binding of cTnI [46,47]. Figure 2 shows the steps and materials used in the assay preparation. In step 1, AuNPs were conjugated with the MGITC reporter molecule and a covalently bound thiol-containing secondary aptamer (5′-thiol-spacer 18-TTTTT CGCAT GCCAA ACGTT GCCTC ATAGT TCCCT CCCCG TGTCC-3′) on the surface. To begin with, 10 μM reduced secondary aptamer was heated in an 85 °C water bath for 5 min and then cooled to room temperature to fold to its tertiary structure. AuNPs were used as the metallic NPs in the aptamer sandwich cTnI assay because they have better stability in the process of conjugating aptamers [48]. Using the method described in previous work [49], 36 nm AuNPs were synthesized and characterized. Specifically, 5.6 μL of 20 μM MGITC was added to 1 mL of the AuNPs (MGITC to NP ratio of 250:1) in a glass vial and shaken for 15 min. Then, 100 μL of 10 μM aptamer was added to the mixture and shaken for 30 min. The mixture was then left still overnight, followed by adding 50 μL of 1 M NaCl. The mixture was shaken for 3 h, followed by adding 50 μL of 1 M NaCl. The mixture was then left still overnight. The aptamer-conjugated MGITC-AuNP was then centrifuged, washed, and resuspended in 1 mL PBS. In step 2, the waveguide surface was functionalized by the silane APTMS and the linker molecule GA, which enabled the covalent immobilization of the amine functionalized primary aptamer (5′-amine-spacer 18-spacer 18-CGTGC AGTAC GCCAA CCTTT CTCAT GCGCT GCCCC TCTTA-3′) in step 3. To achieve this, clean and dry waveguide substrates were covered by a solution of 0.1% APTMS in ethanol at room temperature for 1 h. Then, the substrates were rinsed in ethanol to remove excess silane and cured for 1 h on a 120 °C hot plate. After cooling, the samples were coated with a solution of 2.5% GA in water for 1 h at room temperature and then rinsed in water and dried in air. The resulting waveguide surface has aldehyde functionality to covalently bind the primary aptamer with the amine group (10 μM). In step 4, 25 μL of 1 ng/mL cTnI solution was mixed with the 50 μL of reporter NPs from step 1 and allowed to incubate for 30 min, such that the aptamer binds with the cTnI in the solution. In step 5, the solution was dropped onto the waveguide surface and incubated for 1 h. With the mixed solution on the waveguide surface, the cTnI-reporter NPs were captured by the primary aptamer immobilized on the waveguide surface. The waveguide was then rinsed with buffer solution, leaving only the cTnI-reporter NPs captured.

### 2.5. Optical Testing System Set-Up

**Figure 3:**
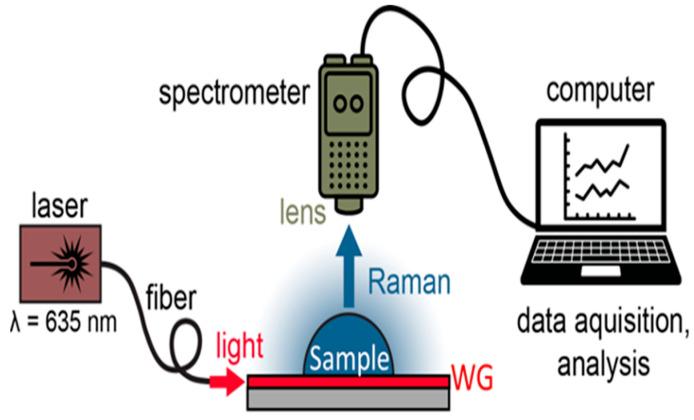
Experimental set-up of the Raman measurements using the optical waveguide device. Light from a λ = 635 nm laser was coupled into a single mode fiber using a reflective lens and then into the waveguide through the butt-coupling method. Raman signals excited by the WG was collected by a handheld Raman spectrometer with a lens adapter attachment.

A test station, pictured in Figure 3, was built to characterize the waveguide performance and was used for all experimental verification steps in **V1**, **V2**, and **V3**. The light from a laser (LSR635CP-2W, Civil Laser Industries) centered at λ = 635 nm with a 5 nm linewidth and 2 W maximum power was coupled into a 3.5 μm core single mode silica fiber through a reflective lens. The excitation light was then butt-coupled into the cleaved front facet of the waveguide, with alignment between the fiber and waveguide monitored via an overhead microscope. Raman emission excited by the waveguides was collected and measured by a compact handheld Raman spectrometer (IDRaman mini, Ocean Optics currently Herisau, Switzerland Metrohm, Herisau, Switzerland) placed above the waveguide device. Spectra were acquired using the point-and-shoot adapter collection optics with NA = 0.50 and working distance of 8 mm, covering the 400 to 2300 cm^−1^ spectral range with a spectral resolution of 15 to 20 cm^−1^. The Peak software (version 1.3.68, Snowy Range Instruments) was used for data acquisition. To perform sensing measurements, 1 μL of the analyte solution was pipetted directly onto the surface of the waveguide device, which was cleaved to a length of 1 cm, such that the entire surface was wetted. Deionized water was used to rinse in between measurements, where the removal of previous substances from the device surface was verified using the handheld Raman spectrometer. This measurement set-up has been demonstrated previously in another work [50]. The measurement conditions for all experiments consisted of 3 sequential acquisitions with an acquisition time of 5 s. All analyses of the Raman signals were performed with Origin software, version 2022, including baseline correction and smoothing via the Savitzky–Golay filtering method to remove the background and noise.

## 3. Results and Discussion

### 3.1. Nanoparticle Characterization

**Figure 4:**
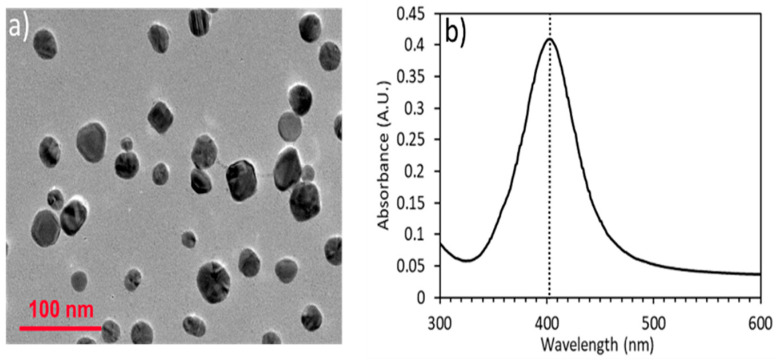
(**a**) TEM image of synthesized AgNPs with an average diameter of 28 nm, (**b**) UV-Vis spectrum showing a peak at λ = 403 nm, corresponding to the LSPR.

**Figure 5:**
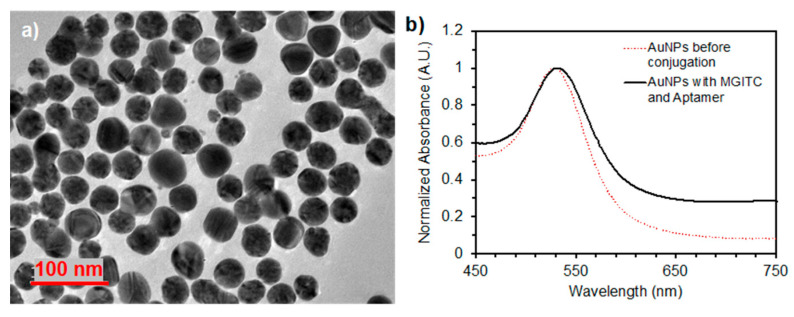
(**a**) TEM image of synthesized AuNPs with an average diameter of 36 nm, (**b**) UV-Vis spectra of the AuNPs before and after aptamer conjugation.

Transmission electron microscopy (TEM) images were acquired on a JEOL JEM-2010 (JEOL, Tokyo, Japan) of both the AgNPs and AuNPs showing the diameter of the AgNPs to be 28 nm and the diameter of the AuNPs to be 36 nm (Figure 4a and Figure 5a). Absorbance spectra of the AgNP colloid were measured on a Tecan Infinite 200 Pro (Tecan, Switzerland) microplate reader for the AgNPs prior to labeling with the Raman reporter dyes and before and after aptamer conjugation of the AuNPs. The absorbance maximum corresponding to the localized surface plasmon resonance (LSPR) was located at a wavelength around λ = 403 nm for AgNPs (Figure 4b) and at 528 nm and 532 nm for the AuNPs and AuNPs conjugated with MGITC and aptamer, respectively (Figure 5b). A ~4 nm peak shift was due to the aptamer and MGITC conjugation that changed the refractive index around the particle. The concentration of the particles was measured with a Nanosight Nanoparticle Tracking Analysis (NTA) system (Malvern, UK) and found to be 2.3 nM. The zeta potential of the particles was measured with a Zetasizer Nano ZS90 (Malvern, UK) and found to be −32.3 mV.

### 3.2. Waveguide Device Characterization and Modeling

**Figure 6:**
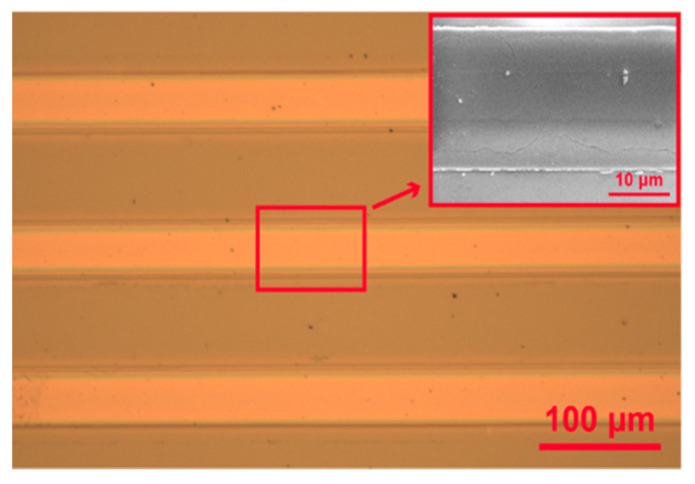
Optical image of AlN ridge waveguides. Inset shows an SEM image of a single waveguide with straight edges and a smooth surface.

The structure of the on-chip waveguide device was examined with optical and scanning electron microscopy (SEM) (MIRA3, TESCAN, Tempe, AZ). Figure 6 presents the top view of an array of AlN strip waveguides with the SiO_2_ undercladding layer. The inset SEM image highlights a single waveguide structure. The width of the waveguide is measured to be 25 μm, and visual inspection from high-magnification SEM shows the structure is defined clearly with a smooth top surface without bending or distortions at the edges. Composition and uniformity of the AlN film has been reported previously [50,51]. The optical waveguide modes of the device were calculated at the λ = 635 nm using the two-dimensional FDM. The waveguide layer used for the simulation had a height of 600 nm, and the refractive indices for the waveguide material and SiO_2_ substrate were **n_WG_** = 2.0 and **n_SiO2_** = 1.5, respectively. Evaluation of the mode profile is crucial in the development of on-chip waveguide-assisted SERS sensors, as the device sensitivity depends on the strength of the evanescent field above the surface, enabling efficient excitation of Raman scattering from the analyte.

**Figure 7:**
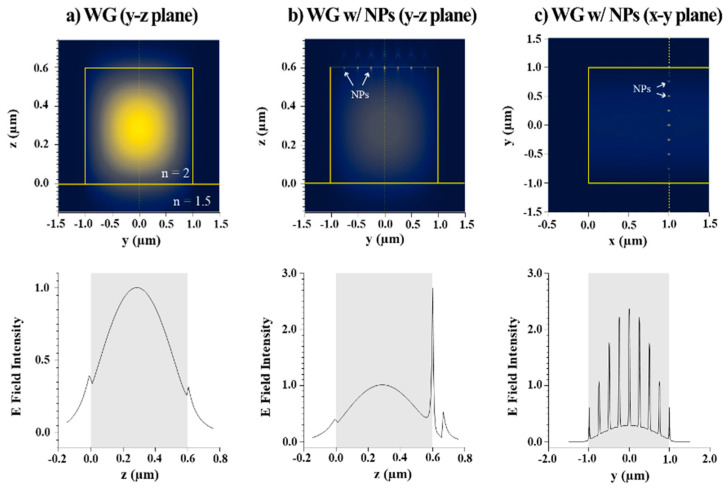
The 2D cross-sectional (y-z plane) profile of the optical field distributions for (**a**) a waveguide alone and (**b**) a waveguide with plasmonic NPs on the surface. (**c**) The top view (x-y plane) of the NPs on a waveguide. Below are plots of the 1D intensity distributions taken from (**a**,**b**) at y = 0 μm and (**c**) at x = 1 μm, as indicated by the dashed line.

Figure 7a depicts an elliptical fundamental mode in the y-z plane for a waveguide without NPs and its field distribution along the z-axis. Clearly, the majority of the field is confined within the waveguide layer at 0 µm < Z < 0.6 µm. A weak evanescent field was extended into the air at Z > 0.6 µm and inside the SiO_2_ undercladding at Z < 0 µm. On the other hand, when the metallic NPs were placed on the waveguide surface, the evanescent field was localized. The y-z and the x-y cross-sectional images of Figure 7b,c show the fields strongly concentrated at the NPs’ positions. To better visualize the field profiles, one-dimensional intensity distributions along the y and z directions are also displayed in Figure 7. The evanescent field was redistributed and amplified by the NPs due to the LSPR. The enhanced field effectively excited Raman signals from the Raman reporter attached to the NPs. Therefore, our NP-on-waveguide device enabled a higher Raman efficiency compared to the devices applying NPs or waveguides alone due to multiple mechanisms taking place simultaneously, including LSPR, wave-guiding, and NP immobilization and trapping on the waveguide surface.

### 3.3. NP-on-Waveguide Characterization

**Figure 8:**
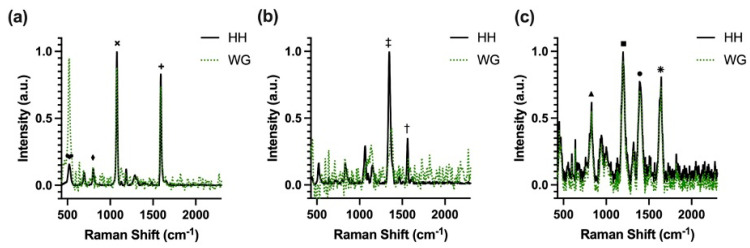
SERS spectra of (**a**) 1 mM 4-MBA, (**b**) 1 mM DTNB, and (**c**) 0.5 µM MGITC acquired using the AgNP-on-waveguide platform (WG) and the handheld spectrometer (HH) configurations. Assigned characteristic features of each compound are marked.

Applying the **V1** configuration. Figure 8 presents the collected SERS spectra of (a) 4-MBA, (b) DTNB, and (c) MGITC using the AgNP-on-waveguide platform and the built-in laser of the handheld spectrometer (HH). The characteristic Raman peaks resolved by the WG device were identified and the results were consistent with the HH configuration and a bench-top Raman spectrometer.

The spectrum of 4-MBA has two strong Raman bands at 1079 cm^−1^ (×) and 1588 cm^−1^ (+), both corresponding to the vibrations of the aromatic ring present in the molecule. Additionally, peaks were observed at 520 cm^−1^ (♥) and 851 cm^−1^, corresponding to the C-S vibration (⧫) and COO- bending, respectively. These peaks were not found in the 4-MBA without NPs due to the relaxation in Raman selection rules known to occur with proximity to a metallic surface [52,53,54]. Characteristic peaks of DTNB can be observed at 1333 cm^−1^ (‡) and 1553 cm^−1^ (†), assigned to symmetric stretching of the nitro group (-NO_2_) and C-C stretching of the aromatic ring, respectively [55,56,57]. The MGITC spectrum can be identified by prominent features at 1618 cm^−1^ (⁕) and 1365 cm^−1^ (●), which are both assigned to stretching of the phenyl-nitrogen (Ph-N) bond and C-C bonds of the aromatic ring. Other identifiable modes include the strong band at 1174 cm^−1^ (■), attributed to an in-plane benzene vibration, and a band at 800 cm^−1^ (▲), corresponding to out-of-plane bending motions of hydrogens on the aromatic ring [58,59]. These results show the ability of our chip-scale waveguide-assisted SERS system to resolve peaks from multiple Raman reporter molecules.

**Figure 9:**
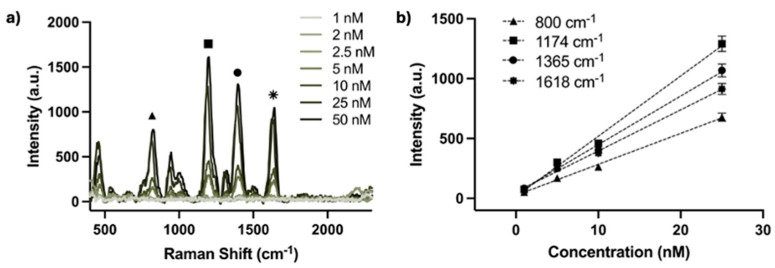
(**a**) SERS spectra of various MGITC concentrations in AgNP solution acquired by the waveguide device, where the characteristic Raman peaks are labeled. (**b**) SERS intensities of the characteristic spectral features versus the concentration of MGITC in AgNPs solution, each shown over the portion of concentration regime with linear response.

To test the performance of the device in quantitative analysis, AgNP solutions with different concentrations of MGITC (1 nM, 2 nM, 2.5 nM, 5 nM, 10 nM, 25 nM, 50 nM) were prepared according to the previously described methods. The testing set-up and experimental parameters, including excitation power and integration time, were kept the same from the initial sensing characterization and maintained throughout the concentration measurements. The SERS spectra of various MGITC concentrations obtained by the waveguide device is shown in Figure 9a. The most intense characteristic peaks, located at 800 cm^−1^ (▲), 1174 cm^−1^ (■), 1365 cm^−1^ (●), and 1618 cm^−1^ (⁕) were selected for more detailed evaluation. Figure 9b displays the plot of SERS intensity versus reporter concentration at those Raman peaks. There is a linear dependence between the MGITC concentration and the SERS intensity within the concentration range between 1 nM and 25 nM. The low detection limit was found to be 1 nM.

**Figure 10:**
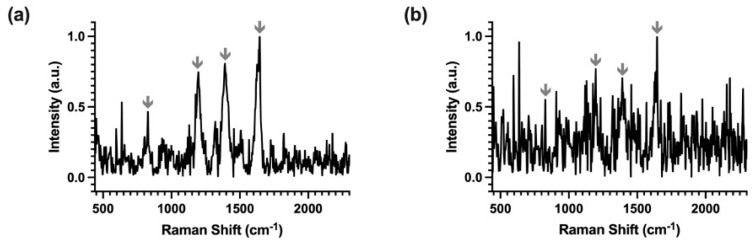
SERS spectra of (**a**) the aptamer-conjugated MGITC-NPs in solution (**V2**) and then (**b**) the captured MGITC-NPs on the waveguide surface applying the aptamer sandwich assay (**V3**). The spectra were collected from the waveguide device with Raman peaks marked.

After the validation in phase **V1** that the waveguide-assisted SERS had good capability of resolving Raman peaks of multiple reporter molecules and generating quantitative signals, the demonstration was extended to the detection of a protein biomarker (i.e., cTnI). Following the procedures described in the phase **V2** and **V3**, MGITC-labeled NPs and the waveguide surface were each immobilized with one of an aptamer pair to capture the cTnI analyte in a sandwich assay design. The same optical test set-up with the waveguide device was used to collect and verify the Raman signal from the aptamer-conjugated MGITC-NP alone in **V2** and then for the detection of the captured cTnI as part of the sandwich assay in **V3**. The SERS spectra from **V2** and **V3** are shown in Figure 10a,b, respectively, with characteristic peaks indicated. The most intense characteristic peaks were the same as those identified in the initial MGITC experiments in **V1** and can be seen in both spectra of the labeled reporter in **V2,** as well as from the captured assay components in **V3**, located around 800 cm^−1^ (▲), 1174 cm^−1^ (■), 1365 cm^−1^ (●), and 1618 cm^−1^ (⁕). These results confirm that the aptamer-conjugated MGITC-NP could generate a strong SERS signal and the addition of the aptamer to the MGITC-NP in **V2** did not alter the Raman signatures of the reporter label. Moreover, these spectral features were also identified in the Raman assay taken after surface washing in **V3**, indicating the successful capture of the cTnI onto the waveguide surface by the aptamer sandwich design.

In the case of the sandwich assay in **V3**, only a thin layer of the aptamer-conjugated MGITC-NP was trapped. However, the Raman intensity is still comparable to the larger volume solution sample when characterizing a droplet of the aptamer-conjugated MGITC-NP solution in **V2**. The well-observed SERS signals from our waveguide-assisted SERS is attributed to the primary aptamer that successfully immobilized on the waveguide surface and its formation of the sandwich binding after capturing the target cTnI protein and the aptamer-conjugated MGITC-NPs. In addition, our Raman spectrum collection configuration has the spectrometer placed above the waveguide surface and perpendicular to the waveguide device, or a ‘top’ collection method. This configuration has the advantage to minimize the background from the excitation laser as compared to other geometries that apply a back collection method [24].

## 4. Conclusions

A waveguide-assisted SERS device using colloidal NPs was developed. Optical on-chip waveguide-assisted SERS was integrated with an aptamer sandwich assay for the detection of the protein biomarker cTnI. Raman intensity was improved through the optimization of waveguide structure and its refractive index. The waveguide evanescent field to excite the Raman signal was further enhanced by the NPs captured on the waveguide surface. The signal enhancement from NPs on a waveguide was shown by modeling and experimental data. Representative SERS spectra from different Raman active compounds, including 4-MBA and DTNB, and chromophore MGITC, were acquired using the waveguide-assisted SERS device. Characteristic Raman peaks associated with different compounds and their vibration modes were identified. A linear dependence between the MGITC concentration and its SERS intensity was observed at 800 cm^−1^, 1174 cm^−1^, 1365 cm^−1^, and 1618 cm^−1^, where a detection concentration of 1 nM was achieved. Effective trapping of the protein and the aptamer–NP complex onto the waveguide surface enabled the detection of the cardiac biomarker cTnI. These results demonstrate that our on-chip optical waveguide assisted SERS miniaturized sensing platform has the potential to detect various analytes from small molecules to large proteins. The top collection method used in this study reduces background from the excitation laser and minimizes the need for additional filters or optical components. However, the Raman scattering collected, and thus the overall signal, is limited by the collection spot size of the Raman spectrometer—only 2.5 mm compared to the active waveguide length in centimeters. Considering this, modification of the Raman collection geometry using a different spectrometer and a back collection method would be expected to increase the effective interaction length and signal intensity by an order of magnitude. In this configuration, resolution would be highly dependent on the waveguide dimension and structure. The concept demonstrated in this work could be extended to a multiplexing analysis, using multiple dyes such as the 4-MBA, DTNB, and MGITC shown to spectrally separate Raman signals from multiple assays in a simultaneous detection method. Similarly, the use of multiple waveguides and other photonic circuit structures could be applied in the design to create different active areas that are spatially separated for multicomponent analysis.

## Data Availability

The data presented in this study are available on request from the corresponding author.
